# Combined physiological and metabolomic analysis reveals the effects of different biostimulants on maize production and reproduction

**DOI:** 10.3389/fpls.2022.1062603

**Published:** 2022-11-23

**Authors:** Bingyan Li, Dali Song, Tengfei Guo, Xinpeng Xu, Chao Ai, Wei Zhou

**Affiliations:** ^1^ Institute of Agricultural Resources and Regional Planning, Chinese Academy of Agricultural Sciences, Beijing, China; ^2^ Institution of Plant Nutrition and Environmental Resources, Henan Academy of Agricultural Sciences, Zhengzhou, China

**Keywords:** plant biostimulants, grain quality, stress resistance, metabolomics, nutrient uptake

## Abstract

Plant biostimulants (PBs) are a potential strategy to improve crop growth and grain quality. In the present study, 100 mg/L trehalose, chitosan, humic acid and gamma-aminobutyric acid treatments were applied to analyze the effects of maize production and reproductive characteristics. The contents of nitrogen, phosphorus and potassium and grain quality were significantly affected by the PBs, but not yield. The seed germination rate of all PB treatments was significantly reduced, but the drought resistance of progeny seedlings was significantly improved, with humic acid having the strongest effect. Liquid chromatography mass spectrometry analysis indicated that the disruption of the tricarboxylic acid cycle, probably due to the blockage of intermediate anabolism, reduced the supply of energy and nutrients in the early stages of germination, thus inhibiting seed germination, while the increased resistance of the offspring seedlings may be due to the up-regulation of the synthesis of unsaturated fatty acids and alkaloids by humic acid treatment. This study revealed the similarity and heterogeneity of the effects of different PBs on nutrient accumulation, yield characteristics and grain quality of maize, providing guidance for the application of PBs in intensive and sustainable agricultural production.

## 1 Introduction

Corn, which was initially domesticated from a weedy species in central Mexico, has become an important cash crop worldwide after 7,000 years of development, with a wide range of uses in food, feed, industrial raw materials and bioenergy (Qi et al., 2012; Ranum et al., 2014). In 2018, global annual corn production was about 1.148 billion tons. China contributed 257.3 million tons (22.4%), second only to the United States, which produced 366.6 million tons (31.9%), making it the world’s second largest corn producer (Cakmakci and Sahin, 2021; Ma et al., 2021). However, feeding 18% of the world’s population on less than 9% of its arable land has become a major challenge for China’s agricultural production (Duan et al., 2021).

It is generally believed that maize yield is mainly affected by environmental factors, including solar radiation, humidity, temperature and nutrients (Duncan et al., 1965). Hussain et al. have shown that drought stress leads to field crop yield losses as high as 30–90% (Hussain et al., 2019). According to Hao et al., too much or too little light intensity reduces the photosynthetic rate and crop yield (Hao et al., 2020). Li et al. indicated that sufficient nutrient supply has positive effects on improving crop photosynthesis and reducing transpiration (Li et al., 2009). These results underscore the impact of even subtle variation in environmental factors at specific developmental stages on crop yield, especially for seedlings that are typically less resistant to stress. Plant biostimulants (PBs) have the potential to play key roles in mediating the effects of climate change and improving sustainability by reducing non-agricultural chemical inputs and helping to enhance plant stability under stress (du Jardin, 2015). PBs are a class of substances distinct from fertilizers, and they are divided into humic substances, protein hydrolysates, chitosan and seaweed extracts, among other categories, based on the plant responses they cause, rather than their composition (Bhupenchandra et al., 2020). Chen et al. showed that humic acid exhibited positive regulatory effects on soil nutrients and plant growth (Chen et al., 2022). Muley et al. showed that 75 mg/L application of chitosan to potato promoted resistance to drought stress while improving growth performance (Muley et al., 2019). Trehalose enhanced the antioxidant enzyme activity of rice under salt stress (Nounjan et al., 2012) and improved the growth of sunflower shoots and roots under drought stress (Kosar et al., 2021). Cut *Anthurium* flowers treated with gamma-aminobutyric acid exhibited improved resistance to chilling injury by modulating antioxidant enzyme activity (Soleimani Aghdam et al., 2016). The simple or complex molecular structures of PBs also imbue them with different functional properties. For example, humic acid, which contains many carboxylic acids, phenols, alcohol hydroxyl groups and other crown energy groups, can be used in sewage treatment (Zhao et al., 2019); the special molecular structure of trehalose is considered to provide a new means of drying food (Roser, 1991). Chitosan has been widely used in the medical field because of its biocompatibility and non-cytotoxicity (Choi et al., 2016). Gamma-aminobutyric acid is widely used in food as a bioactive compound (Zhao et al., 2021). Although these substances have been extensively studied and used in agricultural production, few studies have evaluated the effects of different PBs on corn kernel quality and seed stress resistance.

Foliar spraying experiments were conducted on maize seedlings at the three-leaf stage to determine the effects of trehalose, chitosan, humic acid and gamma-aminobutyric acid on maize. The contents of nutrient elements in maize plants at three district vegetative growth stages (i.e., 6-, 10- and 14-leaf stages), yield traits and grain quality at maturity were analyzed. Next, we conducted a germination test on the collected cornkernels and treated the germinated seedlings with 15%PEG-6000 to explore the resistance of progeny seedlings todrought stress. These results were used to evaluate the potential value of PB application to the grain produced. In addition, we used metabolomics to analyze the metabolite changes in corn grain treated with humic acid to reveal its regulatory mechanisms. This study reveals the effects of different PBs on maize growth and reproduction, and provides guidance for the application of PBs in sustainable agricultural development.

## 2 Materials and methods

### 2.1 Experimental design

Field experiment: The experimental site is located in Pingyuan New District, Xinxiang City, Henan Province, China. The corn variety used in this study, ‘Jundan 20,’ is cultivated by the Academy of Agricultural Sciences of Junxian County, Henan Province and was sown on June 8, 2021. Untreated water was used as the control, and 100 mg/L trehalose (Sigma Aldrich Shanghai Trading Co., Ltd., Shanghai, China), chitosan (Shanghai Aladdin Biochemical Technology Co., Ltd., Shanghai, China), humic acid (Sigma Aldrich Shanghai Trading Co., Ltd.) and gamma-aminobutyric acid (Beijing Solai Bao Technology Co., Ltd., Beijing, China) mixed with 0.1% Tween 20 were sprayed during the three-leaf stage once every 5 days for a total of three times(June 18, June 23 and June 28). The supplementary material provides more information on PBs, which are thought to have minimal effects as carbon sources or nutrients due to their low concentration (Calvo et al., 2014). Additionally, during the PBs treatment, the ambient temperature was 23.96°C, the relative humidity was 58.83%, the wind speed was 2.04 m/s, and the direct radiation was 13627329.18 J/m^2^. The average monthly temperature and rainfall during maize growth are shown in **Figure S1**, and detailed field management practices can be found in our previous reports (Li et al., 2022). Plant samples at the 6-leaf stage (V6), 10-leaf stage (V10) and 14-leaf stage (V14) and grains at the mature stage were collected for subsequent analysis.

Germination test: Three hundred seeds treated with different PBs were randomly selected, disinfected with 10% hydrogen peroxide for 10 minutes, washed three times with deionized water, soaked in distilled water for 12 hours and allowed to germinate under protection from light at 25°C.

Indoor seedling growth test: Germinated seeds from different PB treatments were transferred to nursery pots and placed in a light incubator (60% light intensity, 55% relative humidity, 28°C/22°C day/night, 15/9h light/dark photoperiod). The growth medium was 2:1 vermiculite and nutrient soil, and all treatments were supplemented with water until the three-leaf stage of maize. Half of the samples were under control conditions with a standard water supply, and the other half were treated with 15% PEG-6000 to simulate drought stress (Zhang et al., 2021). Leaves and stems were harvested after 7 days of drought and placed at -20°C for subsequent analysis.

### 2.2 Sample determination

2.2

#### 2.2.1 Nutrient elements and yield

Four representative plants from each growth period (V6, V10 and V14) that were treated with PBs and water were collected, washed several times with deionized water, dried, ground and used for analysis (Cui and Caldwell, 1997). Nitrogen, phosphorus and potassium (units are expressed as g/plant) in shoots were determined by the method of Czekała et al. (Czekała et al., 2020). Six rows of maize were randomly selected from each PB treatment for the determination of grain yield, 1000-grain weight, grain number per ear and weight per ear. The moisture content of corn has been calculated as 14% (Yan et al., 2021).

#### 2.2.2 Percentage of seed germination

Seeds with radicals longer than 1 mm were considered to have germinated (Flórez et al., 2007). Over the course of a 7-day germination trial, the number of germinations per day was counted and then used to calculate the germination rate of seeds. By the fifth day, seed germination was nearly complete, and the number of germinated seeds did not increase thereafter.

#### 2.2.3 Drought resistance index

Malondialdehyde, proline, soluble sugar and total antioxidant capacity (T-AOC) in leaves were measured according to the method of Wang et al. (Wang et al., 2021). The content of jasmonic acid and salicylic acid was determined using an ELISA kit (Qi Yi Biological Technology Co., Ltd., Shanghai, China), in strict accordance with the instructions. Hemicellulose in plant stems is converted to reducing sugars by acid treatment, in which they react with 3,5-dinitrosalicylic acid to form a brown substance. The absorbance at a 540 nm wavelength was measured for the calculation of hemicellulose content. Then, 0.3 g of plant stem tissue was added to 1 mL of 80% ethanol and incubated in a water bathed at 95°C for 20 min, and the supernatant was then discarded after centrifugation several times. Then, the volume was adjusted to 0.5 mL with distilled water, and 0.75 mL of concentrated sulfuric acid was slowly added. After naturally cooling, the supernatant was centrifuged, and the absorbance at 620 nm wavelength was measured for the calculation of cellulose content.

#### 2.2.4 Metabolome analysis

Non-targeted metabolomic analysis of corn kernel samples from the humic acid and control treatments was performed using liquid chromatography-mass spectrometry (LC-MS). Mixtures of four biological replicates were prepared per treatment as quality control samples (QC). To 100 mg of ground corn powder was added 400 μL of extraction solution (methanol: acetonitrile: water, 2:2:1 (*V*:*V*:*V*), containing an isotope-labeled internal standard mixture). This sample was flash frozen in liquid nitrogen and quickly mixed for 30 s after thawing, and this process was repeated two to three times. The samples were sonicated in an ice-water bath for 10 min, allowed to stand at -40°C for 1 h, frozen and centrifuged, after which the supernatants were collected.

Compounds with coefficients of variation less than 30% in QC samples were selected as final identification results. After the format of the raw data was converted using ProteoWizard software (version 3.0.9/G1709), the data were preprocessed using R (version 3.3.2), and substance annotation was performed using a secondary mass spectrometry database. The cutoff value for algorithm scoring was set to 0.3. KEGG and HMDB databases were used for functional annotation and functional enrichment analysis of metabolites.

### 2.3 Data analysis

One-way ANOVA was performed using IBM SPSS Statistics 23 software (IBM Corp., Armonk, NY, USA), and the error was shown as the mean ± standard error. Physiological and biochemical analyses were performed with at least three replicates, while metabolomic analyses were performed with four biological replicates. For metabolites, principal component analysis, partial least squares discriminant analysis, Pearson correlation analysis and cluster heat map analysis were conducted with the R statistical computing environment. The criteria for differential metabolites included variable importance of projection (VIP) of partial least squares discriminant analysis (VIP > 1), fold difference between humic acid treatment and control treatment (FC > 2 or FC < 0.5) and statistical significance (*P* < 0.05).

## 3 Results

### 3.1 Characterization of nutrient accumulation in shoots under PBs

**Figure 1:**
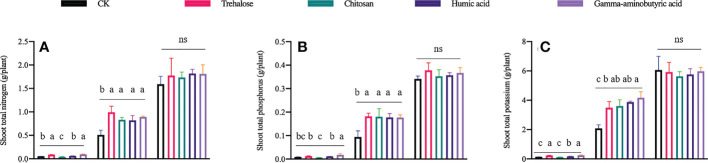
Effects of plant biostimulants on nitrogen, phosphorous and potassium accumulation in maize shoots (**A-C** respectively). Significant differences (P < 0.05) are indicated by treatments labeled with different lowercase letters; ns means no significant difference. Data are presented as mean ± standard error. V6, V10 and V14 represent the 6-, 10- and 14-leaf stage of maize, respectively (n = 4).

For shoots, the accumulation of nitrogen (N) (**Figure 1A**), phosphorus (P) (**Figure 1B**) and potassium (K) (**Figure 1C**) was altered by different PBs during the V6 and V10 stages. Compared with the control at V6, the content of N and K was increased by approximately 67% under the trehalose and gamma-aminobutyric acid treatments; gamma-aminobutyric acid was the only substance that promoted the accumulation of all three assayed nutrients (N, P and K). N was significantly decreased by 21.8% under the chitosan treatment; humic acid treatment only significantly increased the content of K. Compared with the control at V10, all PB treatments significantly promoted nutrient accumulation by about 60.9–99.8%.

### 3.2 Yield characteristics of maize under PBs

**Table 1** shows that the grain number per ear was not affected by PB treatments. Compared with the control, 1000-grain weight and weight per ear were increased by 7.6% and 8.7% under trehalose treatment, respectively; humic acid and chitosan treatments had no significant effect on these two indicators, while 1000-grain weight decreased by 13.2% under gamma-aminobutyric acid treatment.

**Table 1:** Effects of plant biostimulant application on maize yield characteristics.

Treatment group	Grain number per ear	1000-grain weight (g)	Weight per ear (g)	Grain Yield (kg ha^−1^)
Control	464 ± 9.24 a	241.41 ± 0.75 b	105.84 ± 2.07 b	6235 ± 266.05 ab
Trehalose	458 ± 26.56 a	259.83 ± 1.54 a	115.03 ± 3.20 a	6396.25 ± 6.33 a
Chitosan	490 ± 8.08 a	239.40 ± 1.05 b	104.13 ± 2.68 b	5993± 63.34 ab
Humic acid	484 ± 35.51 a	239.09 ± 0.73 b	104.26 ± 2.02 b	5970.73 ± 18.10 ab
Gamma-aminobutyric acid	518 ± 4.62 a	209.66 ± 1.83 c	99.08 ± 1.86 b	5836.35 ± 243.88 b

Data are presented as mean ± standard error values. Significant differences between treatments (P < 0.05) are indicated by different lowercase letters.

### 3.3 Corn kernel quality under PBs

The effects of PBs were significantly altered by nutrient accumulation patterns in grains. Compared with the control, the content of crude protein was significantly increased by 12.2–15.3% by trehalose, chitosan and gamma-aminobutyric acid; the crude lipid content was significantly increased by 24.1% and 10.8% by trehalose and chitosan treatments, respectively. All PB treatments significantly reduced the content of total starch, with the treatments ranked in the following descending order of their effects: chitosan, gamma-aminobutyric acid, trehalose, humic acid.

### 3.4 Germination rate of harvested seeds under PBs

The seed germination rate was inhibited by all PB treatments. Three hundred harvested seeds exposed to different treatments (control, trehalose, chitosan, humic acid and gamma-aminobutyric acid) were subjected to a 7-day germination test. On days 1 and 2, there were no significant differences among all treatments. By the third day, the seed germination rate was significantly inhibited by PBs compared with the control treatment, while there was no difference among different PB treatments. By day 5, the seed germination rates of the control and PB-treated groups were about 96% and 73%, respectively. From the 6th day onward, the remaining ungerminated seeds did not continue to germinate. In addition, owing to the long-term immersion of seeds, mildew began to appear, so the data were not counted thereafter.

### 3.5 Stress resistance of progeny seedlings under drought

**Figure 2:**
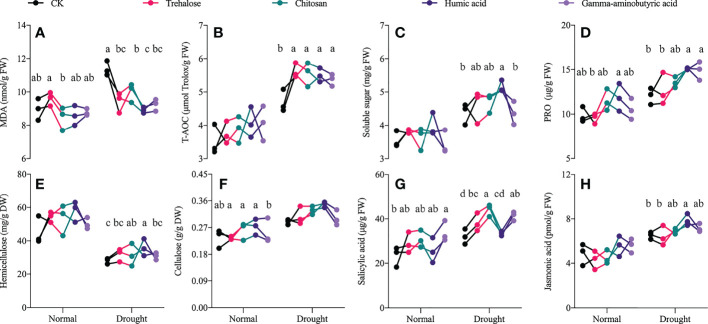
Effects of drought stress on the drought resistance of plant biostimulant-treated progeny seedlings. Significant differences between treatments (*P* < 0.05) are indicated by different lowercase letters. Data are presented as mean ± standard error values (*n* ≥ 3). **(A)** malondialdehyde, **(B)** total antioxidant capacity, **(C)** soluble sugar, **(D)** proline, **(E)** hemicellulose, **(F)** cellulose, **(G)** salicylic acid, **(H)** jasmonic acid.

The application of PBs mainly changed the stress resistance of progeny seedlings under drought stress, rather than affecting the growth of seedlings under a normal water supply. Compared with the control under drought, all PB-treated progeny seedlings showed significantly reduced contents of malondialdehyde (MDA) (**Figure 2A**) by 12–21.7%, but the activity of total antioxidant capacity (T-AOC) (**Figure 2B**) was significantly increased by 14.3–19.6%. In addition, trehalose treatment only changed the content of salicylic acid (**Figure 2G**) in seedlings compared with the control group under drought; hemicellulose (**Figure 2E**) and salicylic acid contents were significantly increased by 13.8% and 37.7% by chitosan treatment, respectively. Proline (**Figure 2D**) and salicylic acid contents in the gamma-aminobutyric acid-treated progeny seedlings were 1.24 and 1.29 times those of the control group, respectively; cellulose content (**Figure 2F**) was not significantly different from that of controls in all PB-treated progeny seedlings. Soluble sugar (**Figure 2C**), proline, hemicellulose and jasmonic acid (**Figure 2H**) were all significantly increased in the humic acid treatment compared to other treatment groups, with the most significant changes observed among all PBs treatments.

### 3.6 Characteristics of metabolic activity in maize grain induced by humic acid

A total of 5271 metabolites were identified by metabolomic analysis by LC-MS under humic acid and control treatments, including 2952 metabolites in positive ion mode and 2319 metabolites in negative ion mode (File S1). Of these metabolites, 3491 were involved in metabolic activities, accounting for 66.2% of the total number of metabolites, and they can be categorized as follows: 1432 global and overview maps, 450 amino acid metabolism and metabolism of other amino acids, 371 chemical structure transformation maps, 308 biosynthesis of other secondary metabolism metabolites, 189 carbohydrate metabolism, 174 lipid metabolism and relatively fewer nucleotide and energy metabolism (**File S2**, **Figure S2A**). Principal component analysis (PCA) showed that the humic acid treatment was significantly separated from the control treatment along principal component 1, and principal components 1 and 2 explained 31.4% and 17.8% of the variance in the data, respectively (**Figure S2B**).

A comparative model of the PLS-DA analysis was applied to analyze the differential regulation of metabolites in grain. The R2Y and Q2Y of the model were 0.981 and 0.637, respectively, explaining 40.69% of the total data variance (**Figure S3A**). The humic acid treatment exhibited a smaller and denser span of principal component 2 values than the control group, indicating that metabolic activity in maize kernels is strongly induced by humic acid. A total of 429 differential metabolites (**File S3**, **Figure S3B**) were screened by analyzing the changes in metabolite abundance. These differential metabolites are mainly lipids, amino acids and carbohydrates, accounting for 8.1% of all metabolites. Among them, the number of up-regulated metabolites was 204, and the number of down-regulated metabolites was 225, as displayed in the form of a heat map in **Figure S3C**.

### 3.7 Metabolic characterization of differential metabolites in humic acid-treated corn kernels

**Figure 3:**
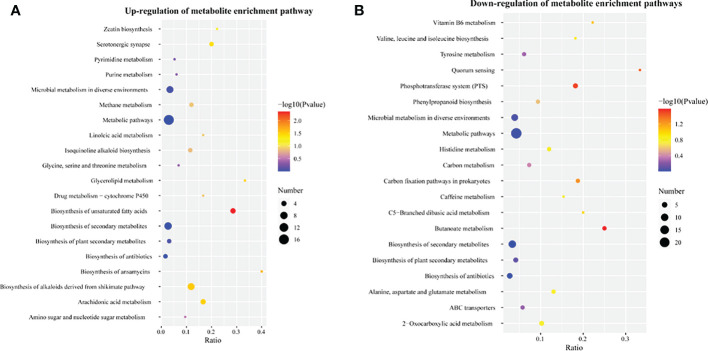
Enrichment analysis of differential metabolites in corn kernels after humic acid treatment. **(A)**, Enrichment analysis of up-regulated differential metabolites. **(B)**, Enrichment analysis of down-regulated differential metabolites. The abscissa is the ratio of the number of differential metabolites to the number of total metabolites identified in the pathway. The color of the dots represents the *p*-value of the hypergeometric test. The size of the dots represents the number of differential metabolites in the corresponding pathway.

Metabolites were functionally annotated using the KEGG database. We performed enrichment analysis for these up- and down-regulated metabolites separately. Up-regulated differential metabolites were mainly enriched for unsaturated fatty acid (map01040) (nervonic acid, dihomo-gamma-linolenate, eicosadienoic acid, tetracosanoic acid were increased by 1.43-4.84 times), biosynthesis of alkaloids derived from the shikimate pathway (map01063) (vindoline, (*S*)-reticuline, protopine, quinidine, tubocurarine, tetrahydroharmine were significantly increased by more than 2.1 times), zeatin biosynthesis (map00908) (uridine diphosphate glucose and 5′-methylthioadenosine were 2.68 and 2.67 times higher than the control, respectively), glycine, serine and threonine metabolism (map00260) (d-glyceric acid was increased 1.73 times) and drug metabolism - cytochrome P450 (map00982) (morphine and 4-hydroxytamoxifen were increased by 12.9- and 1.28-fold, respectively) (**File S3, S4**, **Figure 3A**).

Down-regulated differential metabolites were mainly enriched in tyrosine metabolism (map00350), alanine, aspartate and glutamate (map00250) (succinic acid semialdehyde was decreased by 82.3%), 2-oxocarboxylic acid metabolism (map01210) (l-ornithine, citraconic acid, 2-isopropyl-3-oxosuccinate, oxoglutaric acid were decreased by 50–67.8%), vitamin B6 metabolism (map00750), phenylpropanoid biosynthesis (map00940), histidine metabolism (hercynine and ergothioneine were decreased by 58.4–68.4%) and ABC transporters (map02010) (digalacturonate, ornithine and mannitol were decreased by more than 65.7%) (**File S3, S4**, **Figure 3B**). In addition, the synthesis of some metabolites involved in amino sugar and nucleotide sugar metabolism (map00520) and isoquinoline alkaloid biosynthesis (map00950) was also inhibited.

### 3.8 Regulatory networks of differential metabolites in humic acid-treated corn kernels

**Figure 4:**
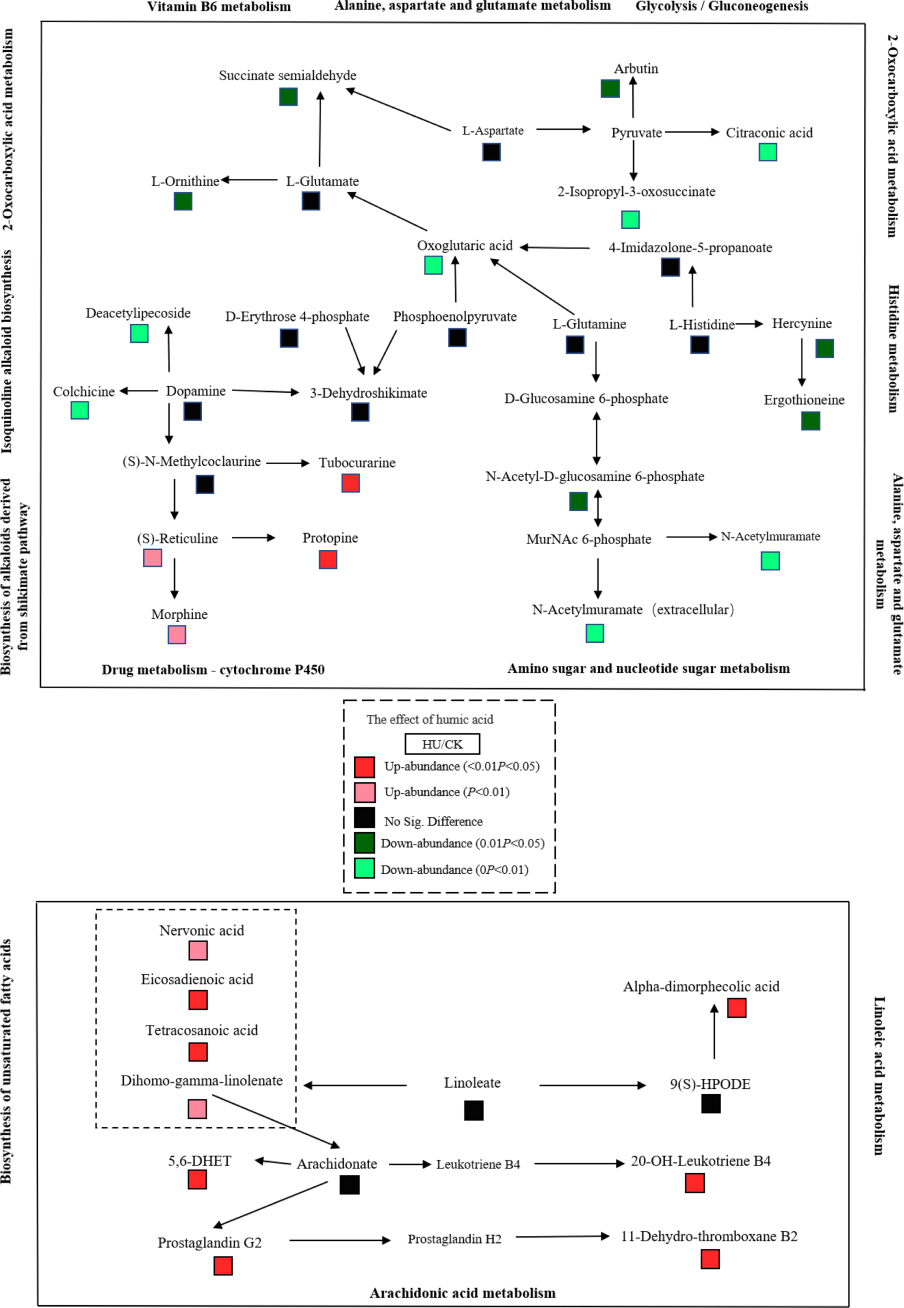
Different metabolites involved in vitamin metabolism, lipid metabolism, amino acid metabolism and carbohydrate metabolism in maize grain between the humic acid and control treatments. Color changes represent the corresponding metabolite abundances. Red, up-regulated abundance (*P* < 0.05); light red, up-regulated abundance (0.01 < *P* < 0.05); black, no significant difference; light green, down-regulated abundance (0.01 < *P* < 0.05); green, down-regulated abundance (*P* < 0.05).

Based on the abundance changes of metabolites, we mapped their regulatory networks in different metabolic pathways (**Figure 4**). Dopamine, oxoglutaric acid and pyruvate, which are located in multiple metabolic pathways. Dopamine causes the down-regulation of deacetylipecoside and colchicine and the up-regulation of tubocurarine, protopine, (*S*)-reticuline and morphine in downstream metabolites. The differential regulation of these metabolites may explain the stability of dopamine content. Compared with the control, the content of oxoglutaric acid was significantly decreased in the humic acid treatment, and its synthesis was bidirectionally regulated by phosphoenolpyruvate l-glutamine and 4-imidazolone-5-propanoate upstream and l-glutamate downstream. Pyruvate was not detected in the metabolome, possibly owing to its high transformation efficiency or low expression abundance. Its regulation resulted in a significant decrease in the abundance of arbutin, citraconic acid and 2-isopropyl-3-oxosuccinate downstream. In addition, we also noticed that arachidonate is the intermediate hub of arachidonic acid metabolism and biosynthesis of unsaturated fatty acids pathways, and its changes are regulated by dihomo-gamma-linolenate in upstream metabolites, 5,6-DHET in downstream metabolites and feedback effects of prostaglandin G2 and leukotriene B4.

Collectively, our results indicate strong interactions among differential metabolites in different metabolic pathways.

### 3.9 Correlation analysis of differential metabolites in corn kernels treated with humic acid

Twenty metabolites were identified based on the *P*-values of the differential metabolites (**Figure S4**). Among them, 12 and 8 metabolites were up-regulated and down-regulated, respectively. These up-regulated metabolites included morphine (meta_pos_1390), uridine diphosphate glucose (meta_pos_2662), adenosine diphosphate ribose (meta_neg_1895) and nervonic acid (meta_pos_1910); the down-regulated metabolites included L-histidine trimethylbetaine (meta_pos_745), trans-aconitate (meta_pos_386), thiamine aldehyde (meta_pos_1125) and arbutin (meta_neg_558), among others. We performed a Pearson correlation analysis (**Figure S5**, **File S5**) of the levels of these differential metabolites, revealing that there were extremely significant correlations among the top 20 differential metabolites in corn kernels treated with humic acid compared with the control. For example, thiamine aldehyde, trans-aconitate and arbutin levels were significantly negatively correlated with nervonic acid levels. Morphine and uridine diphosphate glucose levels were significantly positively correlated with nervonic acid levels. Morphine, arbutin and nervonic acid levels were enriched in the biosynthesis of alkaloids derived from the shikimate pathway, glycolysis/gluconeogenesis and biosynthesis of unsaturated fatty acids pathway, respectively (**File S4**). In addition, the metabolic pathways annotated for these differential metabolites were also identified by enrichment analysis (**Figure 3**), suggesting that they may be important indicators of grain quality.

## 4 Discussion

### 4.1 Effects of PBs on nutrient accumulation and yield characteristics

Because plants are sessile (Liu et al., 2020), in order to sustain growth in variable climates, they frequently adjust their morphological structures and metabolic activities, and plant responses to daily climatic conditions and extreme events exhibit threshold effects (Meza et al., 2008). These changes will have a major impact on global food production. The functional properties of PBs in enhancing plant stress resistance and improving growth have been demonstrated, which means that they may have broad applications in agricultural production. In this study, the accumulation of nitrogen (**Figure 1A**), phosphorus (**Figure 1B**) and potassium (**Figure 1C**) in the shoot of plants were differentially regulated by different PBs at the V6 and V10 stages, but not at V14, compared with controls. These results indicate that there is a time effect of PBs on the promotion of plant nutrient accumulation, which is consistent with our previous findings, that is, the positive effects of PBs on the metabolism and growth of plant shoots were mainly manifested in the early stages of treatment (Li et al., 2022).

Compared with the control, the weight per ear and 1000-grain weight of maize were significantly increased by trehalose treatment, but the yield of maize was not changed by all PBs treatment. This may be due to the weakening or disappearance of the effect of PBs on the nutrient accumulation of maize plants at the V14 stage (the later stage of PB treatments) (**Figure 1**, **Table 1**). In our study, foliar spraying of PBs (100 mg/L) was performed 10, 15 and 20 days after sowing, which to some extent weakened the contribution of nutrient accumulation at the seedling stage of maize to yield formation. Çelik et al. sprayed humic acid (0.01%) at 20 and 35 days after sowing to promote the accumulation of nutrients and dry matter in maize (Çelik et al., 2010). Ibrahim and Abdellatif spraying trehalose at 15,30 and 45 days after sowing increased 1000-grain weight (Ibrahim and Abdellatif, 2016), which is consistent with our results. El-Bassiouny et al. performed two foliar sprays of humic acid (13 mg/L) at 45 and 60 days after sowing and significantly improved the morphological characteristics, mineral content and yield of wheat (El-Bassiouny et al., 2014). This suggests that spraying PBs during the periods of stronger metabolic activity (larger plant size and higher leaf area) seems to be more beneficial for yield improvement. PBs are thought to be rapidly absorbed and transferred by plant tissues (Yakhin et al., 2017), so few studies have discussed the effect of their uptake and loss ratios on crop growth and yield formation. In fact, it is impossible for the foliar sprayed PBs to be fully utilized by the plants, which may exaggerate the amount of PBs to some extent. Therefore, in order to further improve local crop yields, more targeted spraying strategies need to be explored.

### 4.2 Effects of PBs on corn grain quality characteristics

Corn is one of the most economically important crops globally, with more than 35,000 uses (Miao et al., 2006). Maize quality is affected by a combination of factors including genetics, climatic conditions and soil properties, among others. Total starch, crude protein and crude lipid contents comprise most of the corn kernel and are important factors in characterizing the quality of corn (Chen et al., 2016).

**Figure 5:**
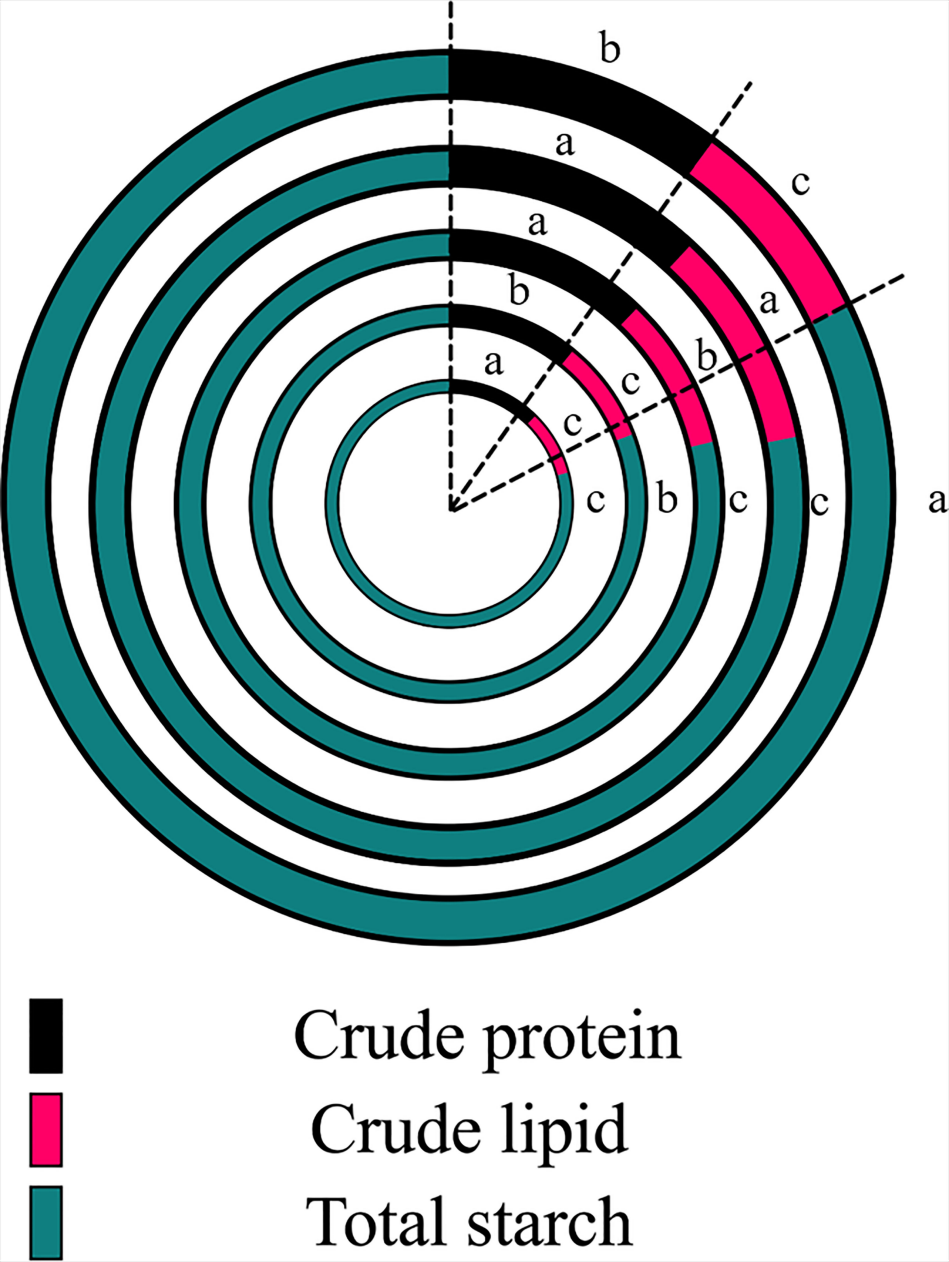
Effects of plant biostimulant application on corn kernel quality based on nutrient content (g/kg). The circles from outside to inside in the figure correspond go the following treatments: control, trehalose, chitosan, humic acid and gamma-aminobutyric acid. Significant differences between treatments (*P* < 0.05) are indicated by different lowercase letters (*n* = 3).

**Figure 6:**
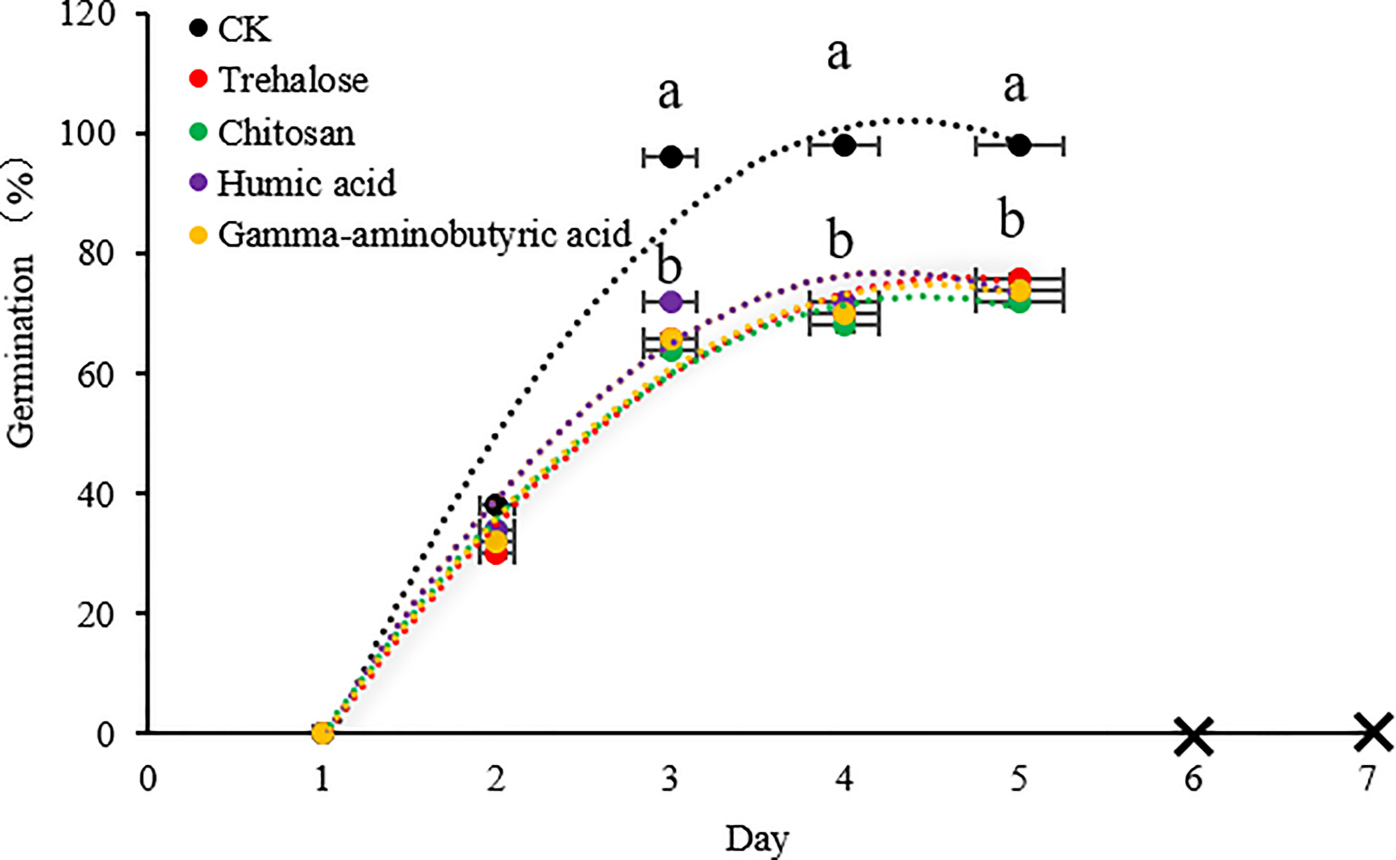
Effects of plant biostimulant application on the germination rate of progeny seeds. No new seeds continued to germinate on days 6 and 7, so data were not collected. Significant differences between treatments (*P* < 0.05) are indicated by different lowercase letters. Data are presented as mean ± standard error values (*n* = 300).

Crude protein and crude lipid were significantly increased by trehalose and chitosan treatment compared to the control treatment. All PB treatments significantly reduced the total starch content of corn kernels (**Figure 5**). Ali et al. reported that the application of exogenous trehalose increased the crude oil content in maize seeds, which exhibited higher levels of drought resistance (Ali et al., 2012). Mukarram et al. showed that chitosan can enhance the metabolic intensity of the source sink by enhancing the uptake and assimilation of minerals by the crop, and ultimately contribute to the improvement of crop yield and quality (Mukarram et al., 2021).This suggests that nutrient accumulation patterns in grains are altered by PBs and are used for growth during the early stages of grain germination. Pairochterakul et al. reported that the germination rate of seeds was negatively correlated with their sugar content (Pairochteerakul et al., 2018), which is consistent with the down-regulation of seed germination rate we observed (**Figure 6**); however, they also stated that using changes in sugar content alone to predict seed germination rate or seed vigor is unreliable. Therefore, we need more in-depth research methods to reveal the effects of PBs on the metabolic activity of maize grains.

### 4.3 Effects of biostimulant application on drought resistance of progeny seedlings

Plant breeding and molecular techniques have driven the improvement of maize varieties and accelerated the commercialization of superior germplasm resources, resulting in greater benefits for producers and consumers (Mazur et al., 1999). After determining that the application of PBs reduced the germination rate of progeny seeds, the stress resistance of progeny seedlings was next studied to evaluate the effect of PBs on seed quality improvement and the value of PBs.

Malondialdehyde (MDA) is a characteristic substance of membrane lipid peroxidative damage (Saidimoradi et al., 2019). Soluble sugars and proline (PRO) are involved in regulating plant osmotic pressure, and similar substances include betaine, which is currently considered by geneticists as a potential mechanism for improving stress resistance in maize (Quan et al., 2004). The metabolic intensity of the progeny seedlings treated with PBs was not significantly different from that of the control treatment under control conditions, but there were significant differences among treatments under drought stress Niu et al. investigated the effect of PBs (foliar spraying at the four-leaf stage) on the growth of tomato seedlings under low and high temperature stress, showing the promotion of aboveground growth by PBs, especially under stress (Niu et al., 2022). This suggests that the application of PBs conferred resistance to environmental stress in the offspring. All PB treatments significantly increased total antioxidant capacity (T-AOC) (**Figure 2B**) and decreased MDA content (**Figure 2A**) in seedlings. The humic acid treatment induced the highest levels of soluble sugar (**Figure 2C**) and PRO contents (**Figure 2D**) compared to the other treatments. A field trial of humic acid treatment conducted by Chen et al. showed that humic acid treatment enhanced drought resistance by modulating maize photosynthetic carbon metabolism, hormones and osmo-regulatory substances (Chen et al., 2022). Cellulose and hemicellulose in polysaccharide polymers are important components of plant cell walls and play an indispensable role in resisting cell wall deterioration during the long course of evolution (Silveira et al., 2013). Compared with the control treatment, both chitosan and humic acid treatments significantly increased the hemicellulose content of seedlings (**Figure 2E**), but there was no significant change in cellulose content (**Figure 2F**). In addition, drought stress significantly decreased hemicellulose content compared with the control water treatment, although this was not the focus of our investigation. These results suggest that cell wall components are altered by PBs; this down-regulation of hemicellulose resource allocation may be utilized for cellulose synthesis, thereby modulating plant stress resistance to abiotic and biotic stress and potentially altering biomass resource utilization efficiency (Juge, 2006; Tiwari et al., 2018). Humic acid treatment significantly increased the content of jasmonic acid (**Figure 2H**) in progeny seedlings under drought stress, but had no significant effect on salicylic acid content (**Figure 2G**). Nguyen et al. (Nguyen et al., 2009) and Sofy et al. (Sofy et al., 2020) determined that jasmonic acid and salicylic acid are stress signaling molecules of abiotic stress and that both exogenous addition and endogenous expression can help enhance the stress resistance of maize to chilling damage and lead poisoning. These results underscore that humic acid enhances the drought resistance of progeny seedlings.

### 4.4 Metabolic mechanism of humic acid reducing the germination rate of maize seeds

Metabolites are key participants in plant stress responses, and their content changes are considered to be the ultimate manifestation of plant responses to environmental stress (Zhang et al., 2016). Regulating the content of beneficial metabolites in grain is an effective strategy to improve maize germplasm resources. However, these metabolites usually have low abundance (<5%) (Chen et al., 2016). Metabolomic analysis can be used to reveal the response mechanism of maize kernels to PBs. All PBs tested in the present study significantly reduced the germination rate of seeds (**Figure 6**), and humic acid was the most obvious treatment for improving the drought resistance of progeny seedlings among all PB treatments (**Figure 2**). Therefore, we chose humic acid and control treatments for liquid chromatography mass spectrometry (LC-MS).

The tricarboxylic acid cycle (TCA) is a central pathway linking almost all individual metabolic activities and is generally considered to be responsible for the oxidation of respiratory substrates and the synthesis of ATP in energy metabolism (Sweetlove et al., 2010). The enrichment analysis of differential metabolites (**Figure 3**) revealed that alanine, aspartate and glutamate metabolism, 2-oxocarboxylic acid metabolism, histidine metabolism and secondary metabolite synthesis pathways were significantly downregulated, thus reducing the energy and substrate supply for protein and enzyme synthesis in the early stage of germination (Zhao et al., 2022). The synthesis of GABA is mainly completed through the GABA shunt pathway, which is an important part of the TCA cycle. The study by Chung et al. showed that the content of GABA gradually increased with the germination of barley seeds, and this rapid increase was dependent on the participation of various amino acids and proteins (Chung et al., 2009). Similar findings also appeared in the germination process of millet seeds (Sharma et al., 2018). GABA provides the carbon skeleton for the TCA cycle, which aids in seed germination (Du et al., 2020). In this study, the content of ornithine, a precursor of GABA synthesis, was significantly down-regulated by 65.7% in humic acid treatment (File S3). Ornithine acts as a precursor for proline synthesis, and Loenders et al. found that lines overexpressing ornithine-δ-aminotransferase exhibited higher proline content and seed germination rates (Roosens et al., 2002). It is suggested that the down-regulation of GABA synthesis caused by ornithine deficiency induces the disturbance of the TCA cycle. Trans-aconitate (53.9% down-regulated) is the trans isomer of cis-aconitate and is formed from citrate, the accumulation of which is associated with 2-oxocarboxylic acid metabolism. Trans-aconitate represents an additional pool of fixed carbon that helps stabilize the tricarboxylic acid cycle (TCA) and is upregulated by some environmental stresses (Urrutia et al., 2021). Oxoglutaric acid is an intermediate product of the TCA cycle and can act as an N donor in the aminotransferase reaction. Its down-regulation (67.8%) may lead to the blocking of carbon and nitrogen metabolism (Devanathan et al., 2014). Pearson correlation analysis (**Figure S5**, **File S5**) showed a significant positive correlation between trans-aconitate, l-histidine trimethylbetaine and arbutin, indicating convergent changes in amino acid metabolism, glycolysis/glycogenesis and 2-oxocarboxylic acid metabolism linked by the TCA cycle. In addition, the vitamin B6 metabolic pathway was significantly downregulated (**Figure 3**), but did not alter the content of pyridoxal 5’-phosphate in grain (**File S2. S3. S4**). Pyridoxal 5′-phosphate is the activated form of vitamin B6, and it was confirmed that the loss of *Smk2*, a key gene in vitamin B6 biosynthesis, leads to embryonic lethality, but has less effect on the endosperm (Yang et al., 2017). This accords well with our findings, as we found no significant difference between offspring seedlings treated with PBs and control treatments under normal water supply (**Figure 2**).

Taken together, our findings suggest that humic acid causes starvation of key metabolites in the TCA cycle. Therefore, reducing the intensity of nutrient cycling and energy conversion processes, inhibiting early seed germination but not affecting the growth of seedlings after germination.

### 4.5 The mechanism by which humic acid improves the resistance of maize progeny seedlings to drought

Germination is the beginning of the life of a new individual plant and probably its most vulnerable stage, during which any small changes in the environment can be fatal to the development of the embryo (De-la-Cruz Chacón et al., 2013). A large supply of nutrients is stored in seeds for embryo growth and secondary metabolites to enable the exchange of substances and physiological adaptation to the external environment.

The presence of substances that confer resistance to biotic or abiotic stresses in plants was identified. UDP-glucose is a substrate for the synthesis of sucrose and polysaccharides and is thought to be a signaling molecule in plants that is involved in plant growth and development (Janse van Rensburg and Van den Ende, 2018). The abundance of UDP-glucose in grains was significantly increased in humic acid treatment (**File S3**). In fact, reduced UDP-glucose content can lead to abnormal vegetative and reproductive growth of plants (Janse van Rensburg and Van den Ende, 2018). Xu et al. showed that UDP-glucose mediated FeSTAR_2_-involved buckwheat resistance to AI stress (Xu et al., 2019). A study by Wai et al. showed that an increase of UDP-glucose synthesis helped to increase the biomass of sugarcane (Wai et al., 2017). However, it does not imply that UDP-glucose can be overproduced, which usually leads to programmed cell death (Zhang et al., 2020). Morphine is a naturally occurring alkaloid involved in the synthesis of cytochrome P450 (CHRISTRUP, 1997), which is the largest family of enzymes in higher plants, where its family members play important roles in the detoxification of organisms. Cytochrome P450 is a multifunctional catalyst for the synthesis of secondary metabolites and antioxidants in plants and responds to various environmental pressures (Pandian et al., 2020). The cytochrome P450 gene encodes the synthesis of enzymes involved in plant growth, development and stress response (Fang et al., 2021), and cytochrome P450 mutants show higher levels of oxidative damage and inhibition of photosynthesis (Magwanga et al., 2019). Tamiru et al. showed that cytochrome P450 confers stress resistance to drought stress in rice (Tamiru et al., 2015), which is consistent with our results. Eicosadienoic acid is the synthetic precursor of arachidonic acid, which is considered to be an evolutionarily conserved signaling molecule that can effectively regulate the stress response network of plants (Savchenko et al., 2010; Yuan et al., 2014). Exogenous application of eicosadienoic acid alleviates oxidative damage to Arabidopsis under drought stress through the action of ABA (Alfosea-Simón et al., 2020). The abundance of eicosadienoic acid was significantly up-regulated in humic acid treatment, thereby reducing the accumulation of malondialdehyde (**Figures 2A**, **4**). Plant cuticle is the last barrier of almost all plant organs and is of great importance in preventing water loss, pollutant deposition, biotic and abiotic stresses (Van Oosten et al., 2016). Cuticle wax levels are accompanied by an increase in plant drought tolerance, and Lü’s study showed that the accumulation of tetracosanoic acid in cuticle wax caused by the mutation of the Arabidopsis ECERIFERUM_9_ gene reduces plant transpiration and improves water use efficiency (Lü et al., 2012). Humic acid may endow corn seeds with initial water absorption by increasing the content of tetracosanoic acid and accelerate the germination process of seeds. Nervonic acid is a long-chain monounsaturated fatty acid that plays an important role in brain development and nerve improvement (Liu et al., 2021). Plant oil sources with high levels of nervonic acid have been gradually developed for medicine and health care products. Accordingly, corn plants treated with humic acid may be a potential way of producing nervonic acid. These alkaloids and unsaturated fatty acids, which were significantly up-regulated in humic acid treatment, accounted for the majority of the up-regulated metabolites in grain. Mohamed et al. found that these up-regulated unsaturated fatty acids and alkaloids enhanced soybean resistance to water deficit (Mohamed and Latif, 2017), and similar results were also shown in a study of interspecific drought resistance in soybean (Wang et al., 2022). In conclusion, these up-regulated metabolites altered the stress resistance of maize grains to a certain extent, reduced the accumulation of malondialdehyde in maize plants during drought stress by activating the antioxidant defense system (**Figure 2**), and improved the growth status of the crops.

PBs are attracting more and more attention from agricultural producers because of their low required application levels, high efficiency, non-polluting properties and significant promotion of plant growth and yield formation. Improving the current status of maize production is important for sustainable and intensive development of agriculture. This study showed that trehalose, chitosan, humic acid and gamma-aminobutyric acid significantly promoted nutrient uptake in the shoots of maize at the early and mid-treatment stages. However, the contribution to the corn yield in the later growth period is small. In addition, all PBs changed the quality of maize grains, which on the one hand, resulted in a significant decrease in seed germination rate and, on the other hand, increased the resistance of maize seedlings to drought stress. Among PB treatments, humic acid treatment performed the best in this regard. Metabolomic analysis of plants under humic acid treatment showed that the synthesis of substances that are intermediates of the TCA cycle was inhibited, leading to disruption of the TCA cycle, which affected the supply of energy and nutrients at the early stages of germination, which may have contributed to the reduction in seed germination, while the increase in drought resistance of the offspring seedlings was mainly due to the large accumulation of unsaturated fatty acids and alkaloids in the seeds, which altered the maize seed quality. In addition, some commercially and medically beneficial substances (nervonic acid and morphine) were further enhanced by humic acid treatment, which may expand the application prospects of maize seeds. In conclusion, this study revealed differential regulatory activities of different types of PBs on crop growth and yield characteristics, and most importantly, they altered seed fertility. This has important implications for the application of PBs in sustainable agricultural production.

## Data availability statement

The original contributions presented in the study are included in the article/**Supplementary Material**. Further inquiries can be directed to the corresponding author.

## Author contributions

DS, TG, XX and CA provided help on sample processing, and WZ supported this research. BL completed the experiment, analyzed the data and wrote the manuscript. All authors contributed to the article and approved the submitted version.

## Funding

The authors gratefully acknowledge funding from the Science and Technology Innovation Project of Chinese Academy of Agricultural Sciences.

## Conflict of interest

The authors declare that the research was conducted in the absence of any commercial or financial relationships that could be construed as a potential conflict of interest.

## Publisher’s note

All claims expressed in this article are solely those of the authors and do not necessarily represent those of their affiliated organizations, or those of the publisher, the editors and the reviewers. Any product that may be evaluated in this article, or claim that may be made by its manufacturer, is not guaranteed or endorsed by the publisher.
