# Supplementary material for: Combined physiological and metabolomic analysis reveals the effects of different biostimulants on maize production and reproduction
Source: Front Plant Sci. 2022 Nov 23;13:1062603. doi: 10.3389/fpls.2022.1062603 (PMC9727306; doi:10.3389/fpls.2022.1062603)
Supplement: Supplementary file 1 [file DataSheet_1.zip › Supplementary Figures.docx]

**Combined physiological and metabolomic analysis reveals the effects of different biostimulants on maize production and reproduction**

Bingyan Li^a^, Dali Song^a^, Tengfei Guo^b^, Xinpeng Xu^a^, Chao Ai^a^, Wei Zhou^a*^

^a^ *Institute of Agricultural Resources and Regional Planning, Chinese Academy of Agricultural Sciences, Beijing 100081, China*

^b^ *Institution of Plant Nutrition and Environmental Resources, Henan Academy of Agricultural Sciences, Zhengzhou, 450002, PR, China.*

**Corresponding Author:**

Wei Zhou: *zhouwei02@caas.cn*

**Supplementary material**

**Materials and Methods**

Trehalose: molecular weight: 378.33, CAS: 6138-23-4, purity: ≥ 99%.

Chitosan: molecular weight: ＜2000, CAS: 148411-57-8, storage condition: room temperature.

Humic acid: CAS: 1415-93-6, quality level: 100.

Gamma-aminobutyric acid: Molecular weight: 103.12, CAS No.: 56-12-2, Purity: ≥ 98.0%, Storage condition: room temperature.

**Supplementary figure**


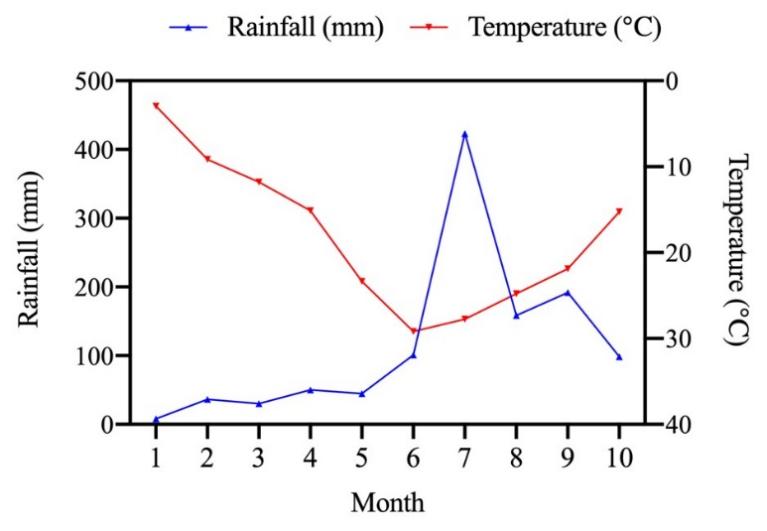


Fig. S1 Monthly mean temperature and rainfall during the growing season of maize.


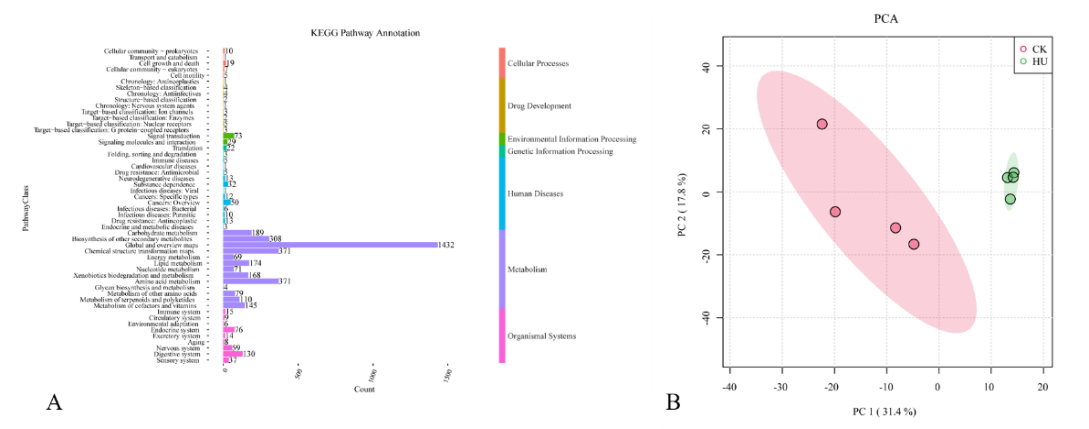


Fig. S2 A: Metabolite information in humic acid and control treatments annotated via the KEGG database. B: Score plot for principal component analysis


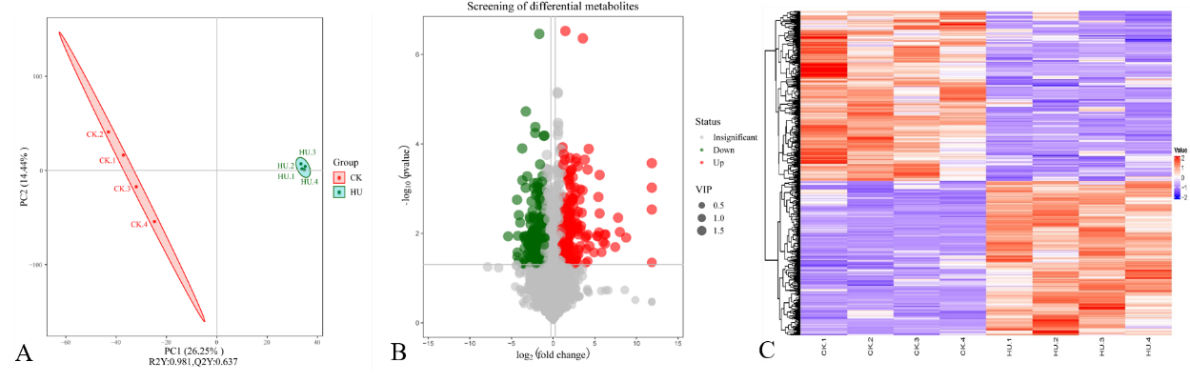


Figure S3. A: Partial least squares discriminant analysis score plot of differential metabolites, B: volcano plot of metabolites, red indicates up-regulation, green indicates down-regulation, and the size of the circle indicates the numerical value of the variable projected importance. C: Cluster heatmap of differential metabolites in humic acid-treated and control-treated groups.


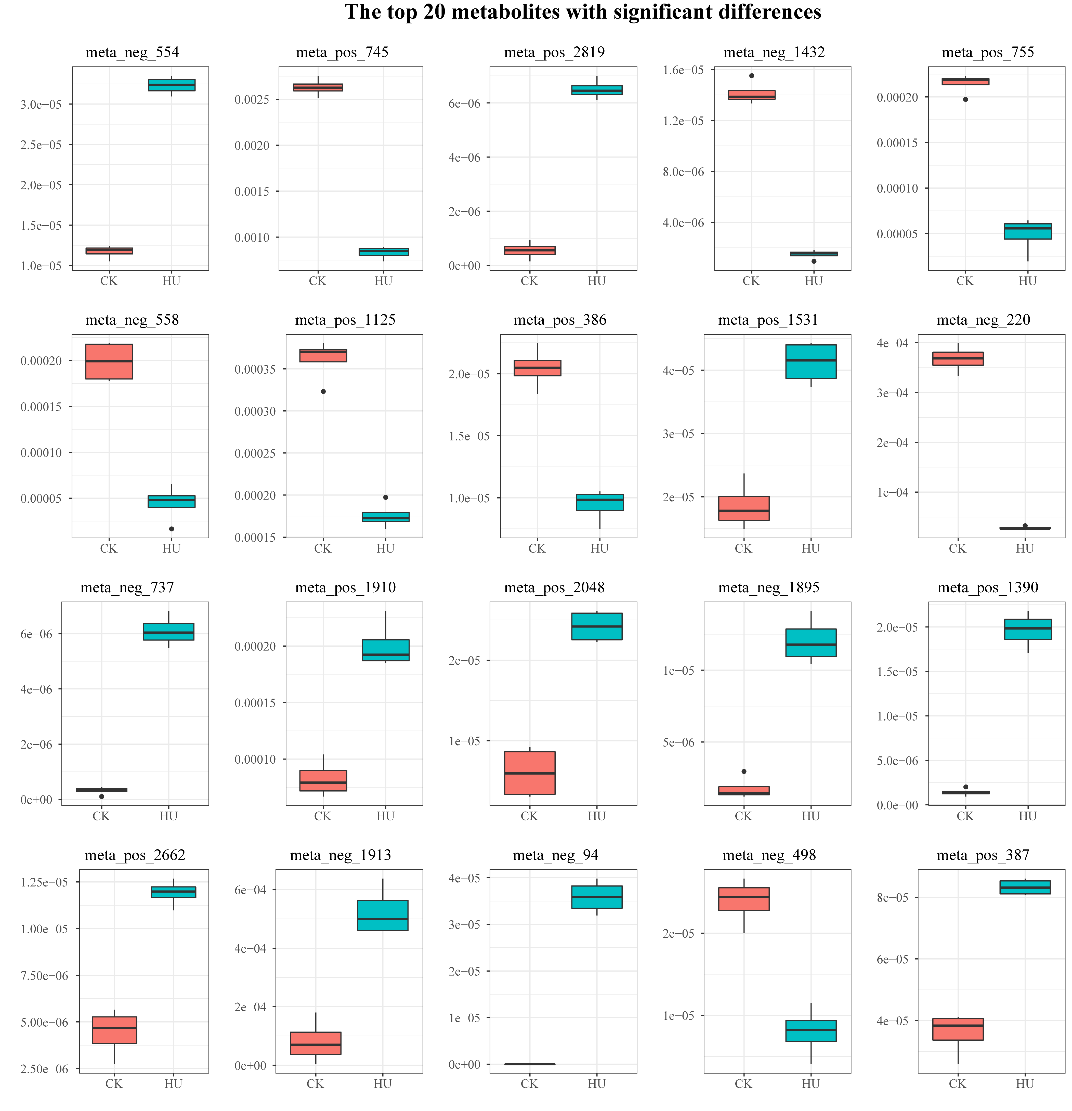


Figure S4. Relative abundance of differential metabolites in the top 20 (ranked by *P*-value) for humic acid and control treatments.


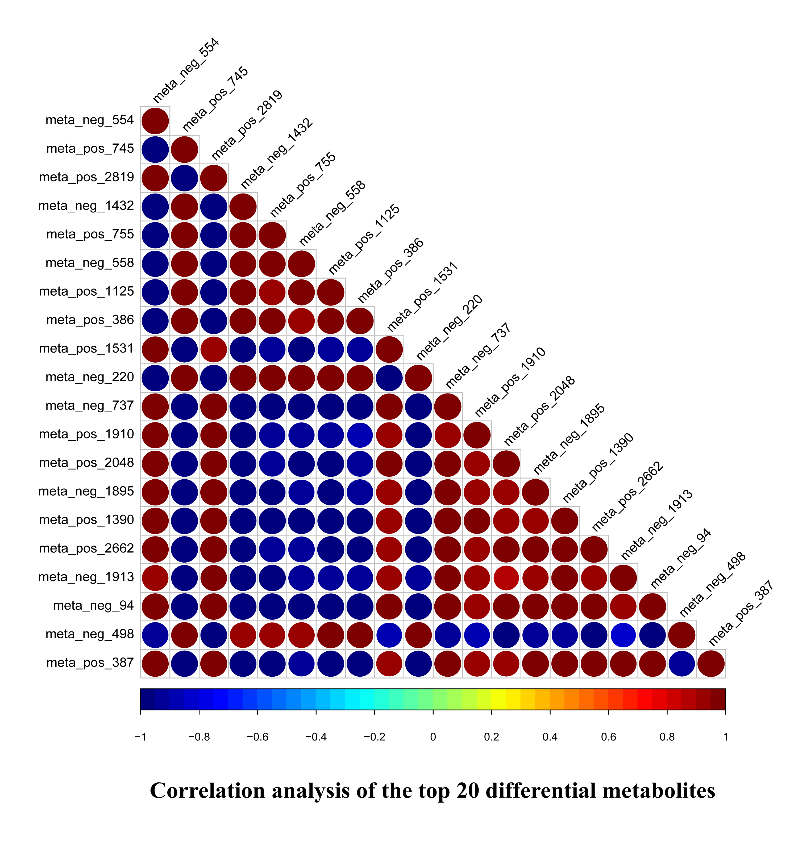


Figure S5. Pearson correlation analysis of the top 20 (ranked by *P*-value) differential metabolites in corn kernels. The significance level threshold was *P* < 0.05. Red, positive correlation; blue, negative correlation.
